# Niel on the Arteries

**Published:** 1845-11

**Authors:** 


### NIEL ON THE ARTERIES.

This work is from the pen of a Philadelphia anatomist whose familiar knowledge of his subject has been aided by the press, the result of which is a volume of great beauty and excellence. Its fine execution commends it to the student of anatomy. It needs no other recommendation.

